# Human biodistribution and radiation dosimetry for the tau tracer [^18^F]Florzolotau in healthy subjects

**DOI:** 10.1186/s41181-024-00259-x

**Published:** 2024-04-02

**Authors:** Kun-Ju Lin, Shao-Yi Huang, Kuo-Lun Huang, Chin-Chang Huang, Ing-Tsung Hsiao

**Affiliations:** 1 Department of Nuclear Medicine, Chang Gung Memorial Hospital, Linkou, Taoyuan Taiwan; 2 Department of Medical Imaging and Radiological Sciences and Healthy Aging Research Center, Chang Gung University, No. 259, Wen-Hua 1St Road, Guishan Dist., Taoyuan City, 333 Taiwan; 3 Department of Neurology, Chang Gung Memorial Hospital, Linkou, Taoyuan Taiwan

**Keywords:** [^18^F]Florzolotau, Alzheimer’s disease, Biodistribution, Radiation dosimetry, Tau PET

## Abstract

**Background:**

Tau pathology plays a crucial role in neurodegeneration diseases including Alzheimer’s disease (AD) and non-AD diseases such as progressive supranuclear palsy. Tau positron emission tomography (PET) is an in-vivo and non-invasive medical imaging technique for detecting and visualizing tau deposition within a human brain. In this work, we aim to investigate the biodistribution of the dosimetry in the whole body and various organs for the [^18^F]Florzolotau tau-PET tracer. A total of 12 healthy controls (HCs) were enrolled at Chang Gung Memorial Hospital. All subjects were injected with approximately 379.03 ± 7.03 MBq of [^18^F]Florzolotau intravenously, and a whole-body PET/CT scan was performed for each subject. For image processing, the VOI for each organ was delineated manually by using the PMOD 3.7 software. Then, the time-activity curve of each organ was acquired by optimally fitting an exponential uptake and clearance model using the least squares method implemented in OLINDA/EXM 2.1 software. The absorbed dose for each target organ and the effective dose were finally calculated.

**Results:**

From the biodistribution results, the elimination of [^18^F]Florzolotau is observed mainly from the liver to the intestine and partially through the kidneys. The highest organ-absorbed dose occurred in the right colon wall (255.83 μSv/MBq), and then in the small intestine (218.67 μSv/MBq), gallbladder wall (151.42 μSv/MBq), left colon wall (93.31 μSv/MBq), and liver (84.15 μSv/MBq). Based on the ICRP103, the final computed effective dose was 34.9 μSv/MBq with CV of 10.07%.

**Conclusions:**

The biodistribution study of [^18^F]Florzolotau demonstrated that the excretion of [^18^F]Florzolotau are mainly through the hepatobiliary and gastrointestinal pathways. Therefore, a routine injection of 370 MBq or 185 MBq of [^18^F]Florzolotau leads to an estimated effective dose of 12.92 or 6.46 mSv, and as a result, the radiation exposure to the whole-body and each organ remains within acceptable limits and adheres to established constraints.

***Trial registration*:**

Retrospectively Registered at *Clinicaltrials.gov* (NCT03625128) on 12 July, 2018, https://clinicaltrials.gov/study/NCT03625128.

## Background

Tau pathology plays a crucial role in neurodegeneration diseases with tauopathy including Alzheimer’s disease (AD), the most common type of dementia (Gale et al. 2018; Hachinski 2019), and non-AD disease, such as progressive supranuclear palsy (PSP), corticobasal degeneration (CBD) (Sengupta and Kayed 2022; Vasilevskaya et al. 2020). Tau positron emission tomography (PET) is an in-vivo and non-invasive medical imaging technique for detecting and visualizing tau deposition within a human brain (Leuzy et al. 2019; Groot et al. 2022).

Recent studies have found that the amount of tau deposition is more associated with disease stages and severity of AD as compared to the accumulation of amyloid-beta plaque (Aß) (Pichet Binette et al. 2022; Hanseeuw et al. 2019). Several tau-PET tracers were developed recently including the first-generation tau-PET tracers of [^18^F]THK compounds (THK5117 and THK5351), [^18^F]AV1451, [^11^C]PBB3 (Harada et al. 2016; Jonasson et al. 2016; Betthauser et al. 2017; Yousefzadeh-Nowshahr et al. 2022; Chien et al. 2014). However, there are some issues related to non-specific binding and sensitivity for the first-generation tau tracers. To solve these problems, some second-generation tracers were developed including [^18^F]MK6240, [^18^F]PI2620 and [^18^F]Florzolotau, also known as [^18^F]APN1607 or [^18^F]PM-PBB3 (Walji et al. 2016; Weng et al. 2020; Mueller et al. 2020).

For the tau tracer [^18^F]Florzolotau, recent clinical studies have shown decreased non-specific off-target binding and a high affinity of [^18^F]Florzolotau imaging (Maruyama et al. 2013; Ono et al. 2017; Hsu et al. 2020).

In Alzheimer's disease (AD) patients, tau pathology predominantly involves the double-helix 4R and 3R tau isoforms. In contrast, the tau pathology in non-AD patients with progressive supranuclear palsy (PSP) primarily exhibits the straight or distorted 4R tau isoform (Goedert et al. 1992; Flament et al. 1991; Saint-Aubert et al. 2017). [^18^F]Florzolotau has been proven capable of detecting both 3R tau and 4R tau isoforms (Ono et al. 2017) and recent investigations have provided compelling evidence for the effectiveness of [^18^F]Florzolotau in identifying tau pathologies in different types of neurodegenerative diseases, such as AD, CBD, PDD, and PSP (Tagai et al. 2021; Li et al. 2021; Miyamoto et al. 2023; Liu et al. 2023a, b; Tang et al. 2023).

The goal of this work is to investigate the internal radiation dosimetry including the absorbed doses within major organs and the effective dose of a single intravenous administration of [^18^F]Florzolotau in healthy adults in the whole body and various organs with the [^18^F]Florzolotau tau-PET tracer.

## Methods

### Participants

A total of 12 healthy controls (HCs) (5 females, 7 males) were enrolled at Chang Gung Memorial Hospital (Age: 56.0 ± 11.8). All participants provided the informed consent before the research procedures. Neuropsychological tests (Mini-Mental State Examination (MMSE), Clinical Dementia Rating (CDR) and CDR scale Sum of Boxes), history and physical examination, electrocardiogram (ECG), and laboratory investigations (blood analysis, urinalysis) were performed before the whole-body PET/CT scan within 30 days. There was no clinically significant abnormality on physical or neurological examinations for each participant.

### Image acquisition: whole-body PET/CT scan

**Fig. 1:**
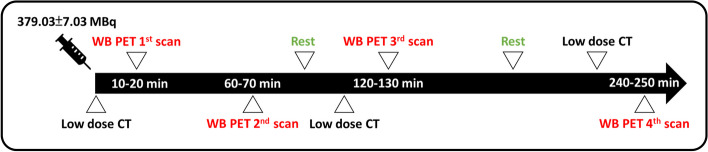
The protocol of whole-body PET/CT scans. Four dynamic whole-body (WB) PET scans were performed for each subject at 10–20 min, 60–70 min, 120–130 min and 240–250 min post-injection

The preparation and synthesis of [^18^F]Florzolotau were performed at the cyclotron facility of Chang Gung Memorial Hospital (Hsu et al. 2020). All subjects were injected approximately 379.03 ± 7.03 MBq of [^18^F]Florzolotau intravenously in order to acquire a better image quality of the whole-body PET and scanned by supine position. Biograph mCT PET/CT (Biograph mCT PET/CT system, Siemens Medical Solutions USA, Inc.) was used for the whole-body PET/CT scans. Images obtained from the low-dose CT scan were applied as attenuation correction maps for [^18^F]Florzolotau (parameters of CT scan: 40 mAs, 120 keV, 512 × 512 matrix, 5-mm slice thickness, 201 slices, 30 mm/s increments, 0.5-s rotation time, the pitch of 0.8). Four cycles of dynamic whole-body PET scans were performed continuously after injecting [^18^F]Florzolotau within 250 min. The scanning time in each cycle was 10 min. Subjects were allowed to leave the scanner before the 3rd and 4th whole-body scans, and the protocol is shown in Fig. 1. A 3-D ordered subset expectation maximization (OSEM) method incorporating the point spread function (PSF) and the time-of-flight (TOF) information with 2 iterations and 21 subsets was used for the whole-body PET image reconstruction (Maeda et al. 2015) with a post-smoothing of a 3-mm full-width at half maximum (FWHM) Gaussian filter (image size: 200 × 200 × 543 mm^3^; voxel size: 4.0728 × 4.0728 × 2.027 mm^3^).

### Image processing

The first step was to manually depict volumes of interest (VOIs) from the co-registered whole-body PETs and corresponding CTs for anatomical information at different time points by using the PMOD image analysis software (version 3.7, PMOD Technologies Ltd, Zurich, Switzerland). The VOIs contained the brain, gallbladder, intestine, stomach, heart wall, kidneys, liver, lungs, spleen, thyroid, bladder, and reproductive organs. The uptake of [^18^F]Florzolotau in each organ was defined by the computation of standard uptake value (SUV) and the equation is shown below:

1 $$SUV = \frac{{decay\,\, corrected\,\, tissue \,\,activity \,\,\left( {Bq/mL} \right)}}{{{\raise0.7ex\hbox{${injection\,\,dose\,\left( {Bq} \right)}$} \!\mathord{\left/ {\vphantom {{injection\,\,dose\,\left( {Bq} \right)} {body \,\,mass\,\left( g \right)}}}\right.\kern-0pt} \!\lower0.7ex\hbox{${body \,\,mass\,\left( g \right)}$}}}}$$

The mean SUV value of $SUV_{organ} \left( t \right)$ within each organ at time t after injection of [^18^F]Florzolotau was assessed in all participants. Then, the mean SUV value was converted into the percentage of injection dose (%ID) for each organ according to the following formula:

2 $$\% ID\left( t \right) = 100\% \times SUV_{organ} \left( t \right) \times \frac{{V_{organ} }}{{M_{WB} }}$$

where %ID(t) is the percentage of injection dose measured at time t post-injection, and $V_{organ}$ and $M_{WB}$ refer to the volume of the organ (ml) and the body mass (g), respectively. In addition, the %ID of the remainder term is defined as ($1 - [\% {\text{ID}}\left( {\text{t}} \right){ }$ within specific organs]).

### Dosimetry calculation

To measure the radiation dose in organs or tissues, the software OLINDA/EXM (Organ Level Internal Dose Assessment, version 2.1) was used for calculating the internal radiation dose (Stabin and Siegel 2018). The workflow of biodistribution estimation complied with the standard method according to Radiation Dose Assessment Resource (RADAR) guide (Stabin et al. 2022a, b). According to the organ %ID at different time points, the time-activity curve (TAC) for each organ was obtained by optimally fitting an exponential uptake and clearance model (Eq. 3) using the least squares method implemented in the OLINDA/EXM 2.1 software. That is, the organ %ID data and corresponding four time points were applied as the input parameters to the OLINDA/EXM software, along with “Data for this organ are decay corrected” option checked. Then, the integration of the TAC (Eq. 4) was calculated as the number of disintegrations occurring in the source organ per given activity, and that is the residence time (Loevinger et al. 1988).

3 $$Activity\left( t \right) = A \times e^{ - a \times t} + B \times e^{ - b \times t} + C \times e^{ - c \times t}$$

4 $$Residence\,\,time = \mathop \smallint \limits_{0}^{\infty } Activity\left( t \right)dt = \frac{A}{a} + \frac{B}{b} + \frac{C}{c}$$

For organs with activities that do not comply with an exponential curve pattern (e.g. gallbladder, stomach), the area under the curve (AUC) was computed by the trapezoidal rule method. The human alimentary tract model (HATM) based on the ICRP100 was applied for the gastrointestinal tract (International Commission on Radiological Protection 2006). Dynamic bladder model was used for the bladder and the voiding intervals of the urinary bladder model is 2.4-h. Then, the OLINDA/EXM 2.1 software was employed to calculate organ-absorbed doses and the effective dose using the residence time of each source organ. This calculation utilized a standard reference phantom adjusted for the individual's specific mass and gender. The simplified equation of the organ-absorbed dose was shown as:

5 $$D = N \cdot DF$$

where *D* is the absorbed dose of the target organ (Gy or rad), and *N* is the residence time (Bq-s/Bq or μCi-hr/μCi), while DF refers to the dose factor including the information of attenuation and the absorption fraction of target organs. Note that DF can be obtained from the Monte Carlo simulation implemented in the OLINDA/EXM 2.1 software (Stabin and Siegel 2018).

## Results

### Demographics of participants

The demographic characteristics of the twelve HCs are shown in Table 1. The age range is from 22 to 66 years, the body weight is 65.3 ± 15.0 kg and the injection dose of [^18^F]Florzolotau is 379.03 ± 7.03 MBq. All subjects exhibit normal cognitive functions, with an MMSE score of 29.25 ± 0.96, and both CDR and CDR-SOB scores of 0. Furthermore, there were no observable changes in the results of the physical examination from both ECG, or laboratory parameters before and after the administration of [^18^F]Florzolotau.

**Table 1:** Demographics of 12 healthy controls

Subject NO	Sex	Age (year)	Height (cm)	Weight (kg)	Injection dose (MBq)	MMSE	CDR	CDR-SOB
1	F	51	154.0	49.0	370.37	30	0	0
2	F	55	153.7	60.1	365.93	29	0	0
3	M	59	167.0	67.5	378.51	29	0	0
4	M	22	177.1	59.0	374.81	30	0	0
5	M	54	161.0	54.9	383.32	30	0	0
6	M	56	176.8	97.6	385.54	30	0	0
7	M	59	164.5	81.5	381.84	29	0	0
8	F	64	152.8	59.3	376.29	27	0	0
9	F	66	171.3	61.0	388.13	30	0	0
10	M	65	172.0	64.4	377.40	30	0	0
11	M	65	175.0	83.0	376.66	29	0	0
12	F	56	156.5	46.0	389.61	28	0	0

*M* Male, F Female, *MMSE* Mini-Mental State Examination, *CDR* Clinical Dementia Rating, *CDR-SOB* CDR scale Sum of Boxes

### Biodistribution of [18F]Florzolotau and time-activity curve (TAC)

**Fig. 2:**
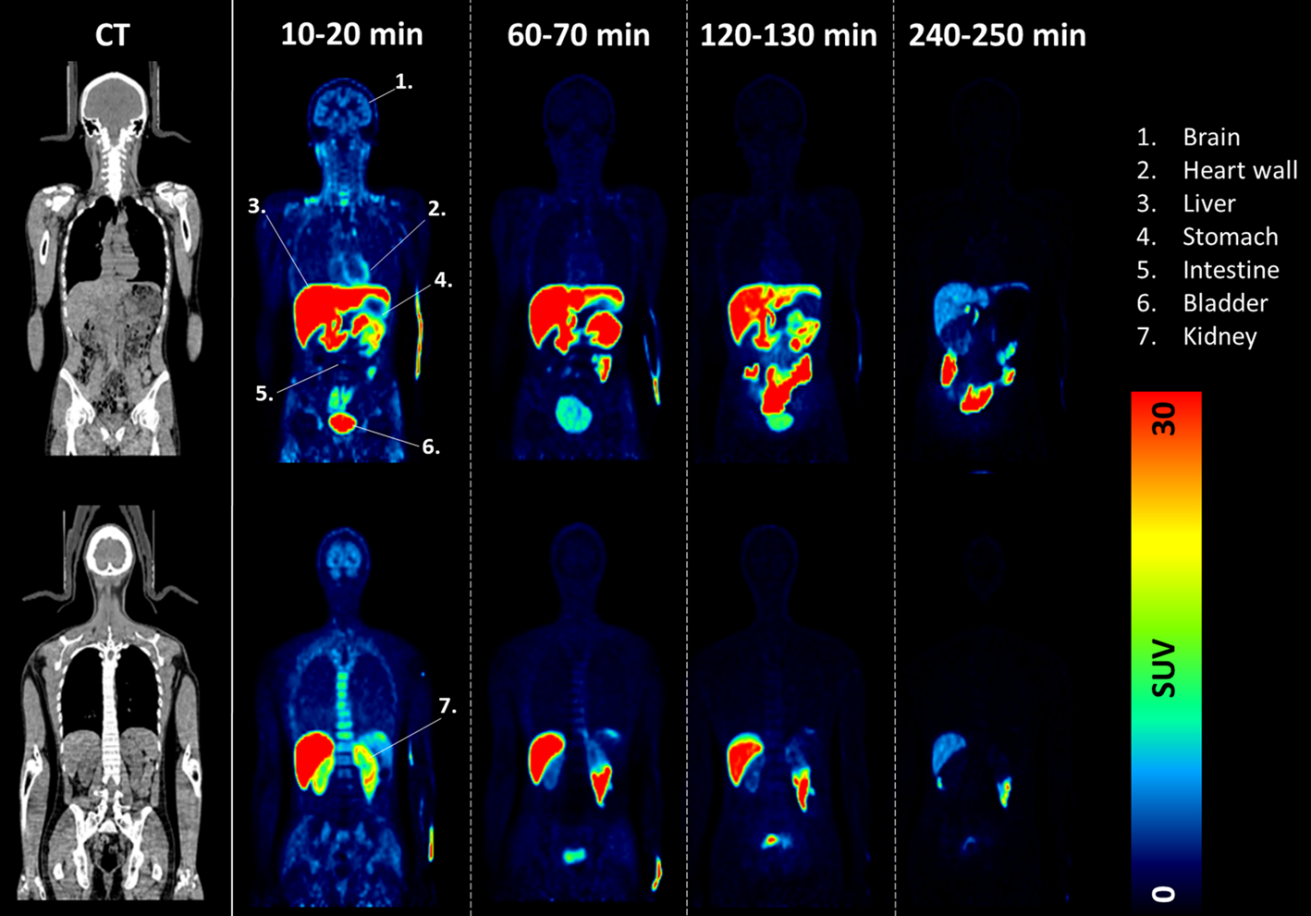
Representative dynamic whole-body PET images (15, 65, 125, and 245 min post-injection) of one healthy control in two coronal views. Images are displayed in the SUV scale

**Fig. 3:**
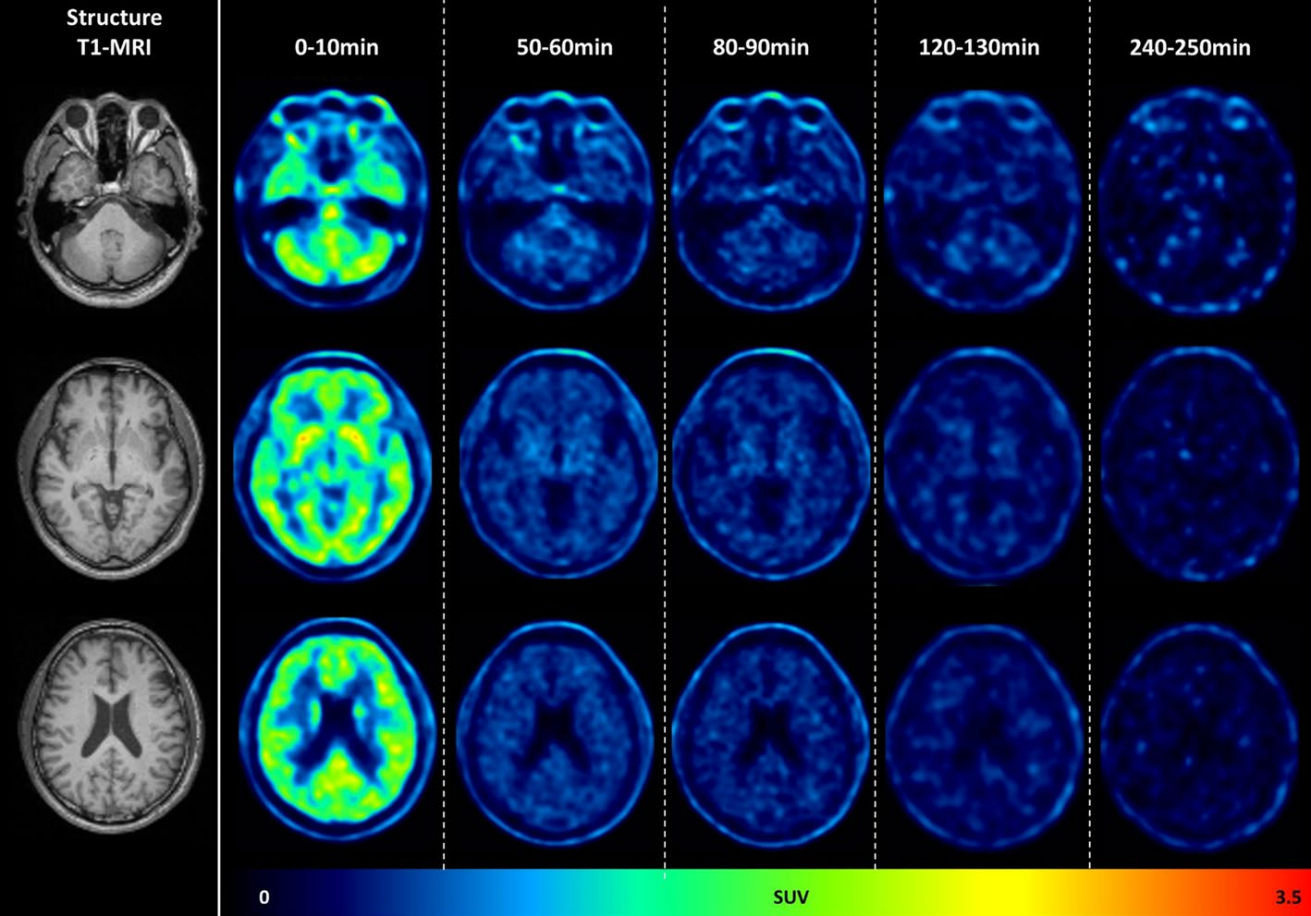
A T1-weighting MRI and dynamic brain PET images of a 65-year-old man. Initially, At the outset, the brain exhibited the highest tracer uptake within the first 10 min post-injection, followed by a gradual decrease in uptake over time, and at 240 min post-injection, there was significant tracer clearance. The left images are T1-MR images in different slices; the right images are corresponding dynamic brain PET obtained at the time intervals of 0–10 min, 50–60 min, 80–90 min, 120–130 min, and 240–250 min post-injection of [^18^F]Florzolotau. All PET images are displayed in the SUV scale

Table 2 presents the uptake of radiotracer in each organ at four different time points and Fig. 2 shows the whole-body image of the biodistribution at four time points from a representative subject. The radiotracer exhibited its highest uptake in the liver, which gradually decreased over time, with %ID values decreasing from 29.36 ± 5.18, 26.72 ± 4.11, and 18.88 ± 4.50 to 11.63 ± 3.65. Subsequently, the radiotracer migrated into the intestine, resulting in an increase in %ID over time. In addition to the liver and intestine, the %ID in other organs (such as the brain, lung, spleen, kidney, etc.) remained consistently below 5% at all time points and exhibited efficient clearance over time. As illustrated in Fig. 3, taking the brain as an example, the %ID in the brain decreased from 2.75 ± 0.33, 1.08 ± 0.18, and 0.86 ± 0.16 to 0.54 ± 0.11.

**Table 2:** The uptake of [^18^F]Florzolotau in each organ at different time points

Organs	15 min	65 min	125 min	245 min
Brain	2.75 ± 0.33	1.08 ± 0.18	0.86 ± 0.16	0.54 ± 0.11
Thyroid	0.13 ± 0.04	0.07 ± 0.02	0.05 ± 0.01	0.03 ± 0.01
Heart wall	1.30 ± 0.68	0.65 ± 0.43	0.53 ± 0.37	0.39 ± 0.32
Lung	4.40 ± 2.79	2.73 ± 1.41	2.22 ± 1.02	1.52 ± 0.65
Liver	29.36 ± 5.18	26.72 ± 4.11	18.88 ± 4.50	11.63 ± 3.65
Gallbladder	0.81 ± 0.53	3.89 ± 5.42	4.96 ± 4.78	1.51 ± 1.46
Stomach	1.44 ± 0.74	1.56 ± 0.92	0.98 ± 0.51	0.40 ± 0.33
Intestine	8.44 ± 2.04	18.75 ± 6.02	31.33 ± 10.43	45.66 ± 7.22
Spleen	0.38 ± 0.28	0.25 ± 0.30	0.18 ± 0.24	0.10 ± 0.12
Kidney	3.26 ± 1.11	2.05 ± 0.73	1.64 ± 0.59	0.94 ± 0.38
Bladder	0.74 ± 0.17	1.61 ± 0.31	0.46 ± 0.37	0.14 ± 0.11
Testis	0.14 ± 0.05	0.13 ± 0.05	0.12 ± 0.05	0.10 ± 0.04
Uterus	0.26 ± 0.05	0.20 ± 0.06	0.18 ± 0.06	0.15 ± 0.08

15 min refers to the average from 10 to 20 min after injection, 65 min refers to the average from 60 to 70 min after injection, and so forth and so on. Units: percentage of injection dose (%ID/organ) represented in mean ± standard deviation

**Fig. 4:**
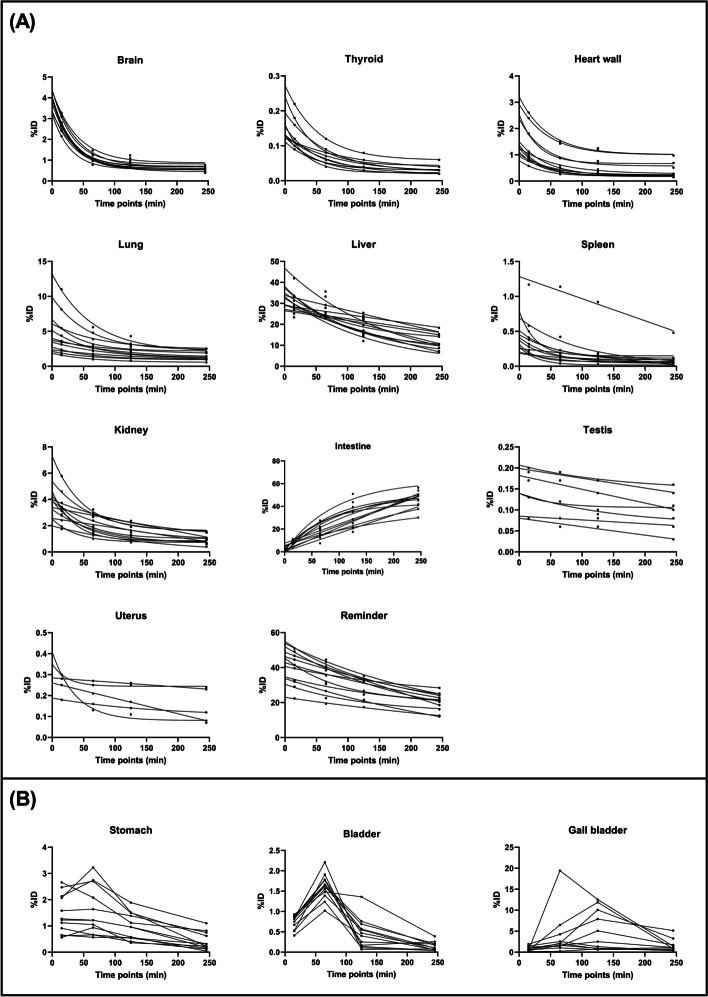
Time-activity curves shown as %ID of each organ across all subjects. All organs TAC in (**A**) were fitted by a nonlinear regression model but not in (**B**)

Figure 4 displays the time-activity curves for each organ in the 12 healthy controls. With the exception of the stomach, bladder, and gall bladder, the %ID in other organs conformed to a nonlinear regression model. Notably, the lung, kidney, and heart wall exhibited rapid wash-in and washout patterns, whereas the liver displayed a gradual uptake and slower washout. The bladder reached a maximum accumulated radioactivity of 2.31 ± 0.31%ID, with a biological half-time of 0.78 ± 0.47 h. The area under the curve reflects the residence time within each organ.

### Organ-absorbed dose and effective dose

The organ-absorbed dose and effective dose are presented in Table 3. The right colon wall received the highest absorbed dose (mean: 255.83 μSv/MBq, CV%: 15.35), then followed by the small intestine (mean: 218.67 μSv/MBq, CV%: 15.15), the gallbladder wall (mean: 151.42 μSv/MBq, CV%: 71.33), the left colon wall (mean: 93.31 μSv/MBq, CV%: 20.69), the liver (mean: 84.15 μSv/MBq, CV: 13.34%) and the kidney (mean: 46.18 μSv/MBq, CV: 24.01%). From both the tissue weighting factors ($W_{T,103}$) and radiation weighting factors ($W_{R,103}$) based on ICRP103, the final effective dose was calculated as 34.9 μSv/MBq with CV of 10.07%.

**Table 3:** Average organ-absorbed dose per unit activity administered and the effective dose

Parameter/Organ	μSv/MBq	CV(%)
Brain	5.81	18.06
Eyes	3.66	23.76
Salivary Glands	4.19	19.39
Thyroid	16.18	21.11
Esophagus	12.38	8.70
Thymus	7.86	10.35
Lungs	17.39	21.79
Heart wall	20.10	32.33
Breasts	6.62	3.00
Liver	84.15	13.34
Gallbladder wall	151.42	71.33
Spleen	18.09	51.31
Pancreas	33.06	10.36
Stomach wall	25.31	25.43
Small intestine	218.67	15.15
Right colon	255.83	15.35
Left colon	93.31	20.69
Rectum	13.65	9.40
Adrenals	28.51	10.53
Kidneys	46.18	24.01
Urinary bladder wall	19.73	18.41
Ovaries	18.36	8.84
Uterus	28.82	12.00
Prostate	10.52	9.42
Testes	16.20	35.28
Red marrow	9.32	16.66
Osteogenic cells	7.21	13.04
Total body	12.89	21.87
Effective dose	34.90	10.07

Voiding intervals of the urinary bladder model is 2.4 h

## Discussion

The objective of this study is to investigate the biodistribution of [^18^F]Florzolotau and to perform whole-body dosimetry calculations using the OLINDA/EXM version 2.1 software. The biodistribution findings reveal that the elimination of [^18^F]Florzolotau primarily takes place through the hepatobiliary and gastrointestinal systems. Initially, the liver exhibits the highest %ID, which gradually decreases over time. Subsequently, the tracer moves into the intestines, leading to an increase in %ID over time. The brain, thyroid, and heart wall exhibit lower %ID levels with effective clearance. Notably, [^18^F]Florzolotau is capable of crossing the blood–brain barrier (BBB), resulting in an initial brain %ID of 2.75 ± 0.33 at 15 min post-injection, followed by gradual clearance. Conversely, nonlinear regression models were not applied to the gallbladder, stomach, and bladder TACs due to anticipated changes in organ volumes over time.

The dosimetry results reveal obvious variations in organ-absorbed doses. The highest absorbed dose is observed in the right colon wall (mean: 255.83 μSv/MBq), followed by the small intestine wall (mean: 218.67 μSv/MBq), gallbladder wall (mean: 151.42 μSv/MBq), left colon wall (mean: 93.31 μSv/MBq), and liver (mean: 84.15 μSv/MBq). Notably, the gallbladder wall's coefficient of variance (CV%) is significantly greater, possibly attributed to factors such as diet and hormone secretion during the examination (Hsiao et al. 2017; Frisch et al. 2019). Using the tissue weighting factors outlined in ICRP103, the computed organ-absorbed doses per single clinical dose of 185 MBq [^18^F]Florzolotau are as follows: right colon wall (2.29 mSv), small intestine wall (0.37 mSv), gallbladder wall (0.25 mSv), left colon wall (0.83 mSv), and liver (0.62 mSv). The cumulative effective dose per 370 or 185 MBq [^18^F]Florzolotau administration for routine clinical application is calculated at 12.92 or 6.46 mSv. Consequently, the radiation doses incurred by organs and the entire body are deemed acceptable and remain within the stipulated constraints as specified in the Code of Federal Regulation (CFR) 21361.1 (Food 2010).

We note that a recent investigation of the radiation dose of [^18^F]Florzolotau, conducted by Miyamoto et al., wherein three normal subjects were examined, encompassing a more extensive dynamic scanning protocol (Miyamoto et al. 2023). In their research, the residence times of the colonic regions were determined through a curve fitting approach. Additionally, %ID values at distinct time points for various colon segments were acquired by directly delineating the volume of interest (VOI). In contrast, our study involved a more limited number of time points. Consequently, we estimated colon residence times by employing the conservative Human Alimentary Tract Model (HATM). In comparison, their computed dosimetry values for the intestines and colons exhibited significant disparities from our results. Nonetheless, the dosimetry calculations for other organs closely aligned with our findings. It's important to highlight that their study's calculated effective dose reached 3.61 mSv per 185 MBq, which is notably lower than our estimated value. The major difference may be possibly due to the number of scanning time points, the use of different methods in estimating the residence times of the gastrointestinal tract, and also the VOI delineation.

In certain biodistribution studies, the gastrointestinal model frequently serves as a tool for estimating residence times within the intestines. To illustrate, Koole et al., in their 2020 study involving the human dosimetry of [^18^F]MK-6240 and utilizing OLINDA ver.1.0, adopted the GI model based on ICRP30. Their findings demonstrated absorbed doses of 46.4 ± 5.5 μSv/MBq for the lower large intestine and 128.0 ± 15.7 μSv/MBq for the upper large intestine (Koole et al. 2020). Similarly, in the biodistribution study conducted by Bullich et al., the GI model in ICRP30 was applied, and their computed dosimetry outcomes indicated absorbed doses of 70.1 ± 7.99 μSv/MBq and 222 ± 28 μSv/MBq for the left and right colon, respectively, among females, and 102 ± 4.16 μSv/MBq and 262 ± 11.7 μSv/MBq for males, respectively (Bullich et al. 2020).

Note that a preliminary result of this study was presented previously in a conference using the OLINDA/EXM 1.1. The calculated effective dose was 34.9μSv/MBq here by using OLINDA/EXM v.2.1, while it was 34.5μSv/MBq by using OLINDA/EXM v.1.1. (Lin et al. 2019). The outcome is similar to the study for the dosimetry calculation of [^18^F]MK-6240, where the calculated effective doses by both versions of the OLINDA/EXM displayed no significant difference (Koole et al. 2020; Ohnishi et al. 2023). Compared to OLINDA/EXM v.1.1, the version of v.2.1 added the choice of the newer phantom, updated special models, and the modified reports of dose factor and tissue weighting factor (Cawthorne et al. 2021). Rather than the phantom used in v.1.1, the newer phantom based on ICRP89 is gender-specific and the modified organ mass provides more accurate anatomic information (Valentin 2002). For the gastrointestinal model, the previous GI model was based on ICRP30, while the updated Human Alimentary Tract Model (HATM) is based on ICRP100, age and sex-dependent. The HATM considers the absorption and retention in other organs or tissues, and the structure is also more complex than the GI model (ICRP30) (International Commission on Radiological Protection 2006).

The effective dose of [^18^F]Florzolotau (34.9 μSv/MBq) is comparable with other tau-PET tracers including [^18^F]PI2620 (33.3 μSv/MBq for an adult female and 33.1 μSv/MBq for an adult male), [^18^F]MK6240 (26.8 μSv/MBq), [^18^F]THK5351 (22.5 μSv/MBq) and [^18^F]AV1451(22.5 μSv/MBq) (Hsiao et al. 2017; Bullich et al. 2020; Ohnishi et al. 2023; Choi et al. 2016). It's important to acknowledge a limitation of this study for the relatively fewer time points in the whole-body PET scans, and that may affect the accuracy of curve fitting, such as yielding a larger area under the curve. However, when confronted with a limited number of time points and the complexity of delineating volume of interests (VOIs) for the intestine, resorting to the gastrointestinal model can serve to estimate residence times within the intestine. Consequently, this study confirmed that the radiation dose of [^18^F]Florzolotau is allowable and within limitations and could be used for disease diagnosis for clinical applications.

## Conclusion

The biodistribution study of [^18^F]Florzolotau showed that the predominant pathways of elimination, encompassing the hepatobiliary and gastrointestinal routes. Subsequent dosimetry calculations have revealed that administering a standard injection of 370 MBq (10 mCi) or 185 MBq (5 mCi) [^18^F]Florzolotau corresponds to an estimated effective dose of 12.92 or 6.46 mSv. As a consequence, the radiation doses incurred by individual organs and the whole-body remain compliant with permissible thresholds and constraints.

## Data Availability

The datasets used and analysed during the current study are available from the corresponding author on reasonable request, Ing-Tsung Hsiao (ihsiao@mail.cgu.edu.tw).
